# French Maritime Pine Bark Extract Alleviates Lung Injury by Regulating Inflammatory–Oxidative–Apoptotic Pathway and P2X7 Receptor Expression in LPS-Induced Sepsis

**DOI:** 10.3390/cimb47090770

**Published:** 2025-09-18

**Authors:** Nergis Ulas, Seckin Ozkanlar, Serkan Yildirim, Omer Aydin, Yunusemre Ozkanlar

**Affiliations:** 1 Department of Internal Medicine, Faculty of Veterinary Medicine, Ataturk University, Erzurum 25240, Turkey; aydinomer@atauni.edu.tr; 2 Department of Biochemistry, Faculty of Veterinary Medicine, Ataturk University, Erzurum 25240, Turkey; seckin.ozkanlar@atauni.edu.tr; 3 Department of Pathology, Faculty of Veterinary Medicine, Ataturk University, Erzurum 25240, Turkey; syildirim@atauni.edu.tr; 4 Department of Pathology, Faculty of Veterinary Medicine, Kyrgyz-Turkish Manas University, Chingiz Aitmatov Campus, Djal, Bishkek 720038, Kyrgyzstan; 5 Department of Internal Medicine, Faculty of Veterinary Medicine, Ondokuz Mayis University, Samsun 55139, Turkey; yunusemre.ozkanlar@omu.edu.tr

**Keywords:** inflammation, lung injury, molecular mechanism, sepsis

## Abstract

Introduction: Sepsis is a dysregulated systemic immune response to infection which may result in mortality. It may also lead to organ injury, including injury to the lung. French maritime pine bark extract (MPBE) has been proposed to prevent/treat various inflammatory diseases due to its strong anti-inflammatory and antioxidant effects. This study evaluates the protective and therapeutic effects of MPBE on lung injury induced by intraperitoneal *E. coli* lipopolysaccharide (LPS) in rats. Materials and Methods: The study design was as follows: Control, MPBE20, MPBE50, LPS, LPS+MPBE20 and LPS+MPBE50. Blood and lung tissue samples were collected 6 h after the LPS induction following a 10-day administration of MPBE. Results: LPS-induced sepsis was confirmed by the overproduction of IL-1β and TNF-α in bloodstream compared to the Control (*p* < 0.001). Lung injury was determined by severe histopathological changes and neutrophil infiltration in the lung tissue following intraperitoneal LPS injection. In lung tissue, MPBE improved the levels of P2X7R, TLR4, NLRP3, IL-1β, TNF-α, JNK, H2AX, 8-OHdG, MDA, GSH, Caspase-1 and Caspase-3, and pathological changes in MPBE+LPS groups compared to the LPS group. Conclusions: MPBE appears to regulate P2X7R signaling and the inflammatory–apoptotic pathway by protecting the lung from oxidative cell damage in LPS-induced sepsis in vivo.

## 1. Introduction

Sepsis is a potentially fatal condition that occurs due to a dysregulated host response to infection, leading to multiple organ dysfunction [1]. The lungs are among the first organs to be affected in sepsis-induced organ injury [2]. Sepsis-induced inflammatory changes in the lung results from the disruption of endothelial barrier integrity and damage to the alveolar epithelium resulting in an acute lung injury (ALI) and a clinical outcome entitled acute respiratory distress syndrome (ARDS) [3]. Lung injury in sepsis causes high morbidity and mortality rates and lacks early diagnostic biomarkers [4]. In *Escherichia coli* (*E. coli*) infection, as a Gram-negative bacterial sepsis, lipopolysaccharide (LPS) activates Toll-like receptors (TLRs) and other cell surface receptors, initiating proinflammatory signaling pathways and cell differentiation, causing inflammatory response in multiple organs, including the lung [5,6]. The immune system produces a cytokine response, a so-called cytokine storm, leading to the release of proinflammatory cytokines, including interleukin-1 (IL-1) and tumor necrosis factor-alpha (TNF-α) which initiate systemic inflammation [7]. Furthermore, sepsis-induced inflammation causes oxidative stress, potentially increasing the levels of free radicals and oxidized compounds within cells. This oxidative damage promotes lipid peroxidation of cell membranes and the accumulation of oxidized proteins, exacerbating the lung injury [8].

Purinergic signaling plays a crucial role in various physiological and pathological conditions [9]. Adenosine 3′-triphosphate (ATP) is abundantly released into the extracellular space from the cellular integrity in response to cellular stress or damage. Excessive levels of extracellular adenosine 3′-triphosphate (eATP) interact with purinergic receptors, particularly with P2X7 receptor (P2X7R), on innate and adaptive immune cells. P2X7R activation plays a crucial role in modulating inflammatory responses, which may be coupled with a high occurrence of apoptotic cell death due to the extreme amount of eATP [10]. P2X7R may also have a critical role in the regulation of caspase-dependent inflammasome activation [11,12]. P2X7R coupling in the apoptotic immune response is partly associated with macrophages [13] and dendritic cells [14]. The activation of the inflammatory to apoptotic pathway has a potential detrimental effect on vital organs such as the lungs during sepsis. Additionally, the P2X7R contributes to the production of proinflammatory cytokines in macrophages stimulated by lipopolysaccharide (LPS) induction [15]. Therefore, P2X7R is a crucial component of the immune response in the development of the excessive inflammatory reaction associated with sepsis and lung injury [16].

A multi-protein complex NOD-like receptor family, pyrin domain-containing 3 (NLRP3) inflammasome plays a key role in the innate immune system by facilitating the maturation of inflammatory caspases, particularly caspase-1, leading to the release of crucial proinflammatory cytokines such as IL-1β [17]. NLRP3 inflammasome activation occurs in two distinct steps. The first step, known as priming, involves the upregulation of NLRP3 and pro-IL-1β expression via nuclear factor-kappa B (NF-κB) activation, which is stimulated by microbial components (e.g., TLR ligands) or endogenous cytokines such as TNF-α [18]. The second phase of inflammasome activation involves the oligomerization of NLRP3, leading to the recruitment and assembly of NLRP3, ASC, and procaspase-1 into a functional inflammasome complex. This process facilitates the conversion of procaspase-1 into its active form, caspase-1, which in turn promotes the maturation and release of proinflammatory cytokines IL-1β and IL-18, amplifying the immune response [19].

French maritime pine bark extract (MPBE) contains several active ingredients, including proanthocyanidins. It is currently available in Europe and North America, where it is used to prevent/treat inflammatory diseases and wound healing with a diverse clinical pharmacology [20]. Previous studies have extensively investigated the molecular mechanism of MPBE with regard to its antioxidant [21,22,23], anti-inflammatory [24,25], anti-cancer [26], and antimicrobial [27] effects in many diseases and organ systems. Furthermore, the therapeutic potential of MPBE has been reported with regard to the management of respiratory diseases such as asthma, rhinitis, chronic obstructive pulmonary disease, and pulmonary inflammation [28,29]. MPBE has been suggested to ameliorate oxidative organ damage and DNA damage due to its strong free radical scavenging and antioxidant properties [30]. Therefore, the aim of this study was to evaluate the effects of MPBE administration on the regulation of the inflammatory–oxidative–apoptotic pathway through P2X7R expression, cytokine response, oxidative stress, apoptotic mediators, and inflammatory changes in lung injury in septic rats induced by intraperitoneal LPS injection, in vivo.

## 2. Materials and Methods

### 2.1. Main Materials

The LPS was purchased from the manufacturer (Lot No: H0522, *Escherichia coli* O55:B5, Santa Cruz Biotechnology Inc., Santa Cruz, CA, USA). First, 10 mg of lyophilized LPS powder was dissolved in 10 mL of 0.9% sterile saline solution. The mixture was then vortexed for 30 min to achieve an appropriate dissolution. The MPBE was purchased from the manufacturer (French maritime pine bark extract, FP09385, Biosynth, Staad, Switzerland). The MPBE used in the study is a standardized product which consist of 96% proanthocyanidins as confirmed by UV–spectrophotometric analysis (Pinus pinaster, Batch No.: 093851701, Certificate of product analysis no.: 89947, Biosynth AG, Staad, Switzerland). MPBE was used in 20 mg/kg and 50 mg/kg dosages for two groups. MPBE was dissolved in 0.9% sterile saline solution and a per os dosage was given to the rats by a daily gavage administration to ensure the accurate quantity per kilogram of body weight for an individual consumption.

### 2.2. Animals

Forty-two male 2-month-old Sprague Dawley rats weighing 150–200 g were recruited in this study. Sprague Dawley rats are among the most widely used outbred albino strain rats that show calmness, ease of handling, and overall availability. Male rats were selected to ensure uniformity in the groups. The animals were randomly divided into 6 equal groups of 7 subjects in each. The animals were kept under standard room conditions with a 12 h light–dark cycle, 21 ± 2 °C room temperature with 55 ± 10% relative humidity and fed with a pellet rat food (Standard rat diet, Bayramoglu Feed and Flour Industry Company, Erzurum, Turkey) and bedding, and ad libitum water. This study procedure was approved by the Local Ethics Council of Animal Experiments (HADYEK Decision No: 2024/3-84) that follows the NIH Guide for Laboratory Animals.

#### 2.2.1. Experimental Design and Treatments

The experimental and treatment procedures were as follows. Animals in the Control group were given 0.1 mL of 0.9% sterile saline solution intraperitoneally and the samples were collected 6 h after the injection. Animals in the MPBE20 group were orally given 20 mg/kg/day MPBE for 10 days [31]. Animals in the MPBE50 group were orally given 50 mg/kg/day MPBE for 10 days [32]. Animals in the LPS group were injected with 5 mg/kg i.p. LPS on day 10 [33]. Animals in the LPS+MPBE20 group were orally given 20 mg/kg/day MPBE for 10 days and 5 mg/kg LPS was injected on day 10. Animals in the LPS+MPBE50 group were orally given 50 mg/kg/day MPBE for 10 days and 5 mg/kg i.p. LPS was injected on day 10. The samples were immediately obtained 6 h after the LPS injection on day 10.

#### 2.2.2. Sample Collection and Analysis

The rats were sedated by mild sevoflurane anesthesia (Sevorane liquid 100%, Abbott Laboratory, Turkey) and blood samples were collected via cardiac puncture on day 10. The lung tissue samples were immediately removed from the thoracic cavity following sacrificing by cervical dislocation. One of the lung lobes was immediately stored for biochemical analysis at −80 °C, while the other lobe was fixed for histopathological examination in 10% formalin by standard processing methods. Blood samples were centrifuged at 1500× *g* for 15 min after coagulation at room temperature for 20 min. The sera samples were stored at −80 °C in standard Eppendorf tubes until analysis.

#### 2.2.3. Serum Cytokine Measurements

Rat specific kits for cytokine analysis were purchased from the manufacturers. TNF-α (Cat No: E0764Ra, BT Lab, Zhejiang, China), IL-1β (Cat No: YLA0030RA, YL Biont, Shangai, China) and IL-8 (Cat No: E1167Ra, BT Lab, Zhejiang, China) were assayed in the sera samples by the solid-phase sandwich ELISA method according to the manufacturers’ instructions. Absorbance measurements were performed using an ELISA reader (BioTek, μQuant, Winooski, VT, USA).

#### 2.2.4. Determination of Oxidative Stress Markers in Lung Tissue

Lung tissue samples were prepared to measure the concentrations of malondialdehyde (MDA) and glutathione (GSH) for each sample. Briefly, liquid nitrogen was added to the lung tissue. The tissue was lysed using a tissue lyser (Tissue Lyser II, Qiagen, Hilden, Germany) for 3 min. The sample containing PBS at pH 7.4 was then homogenized at 30 Hz for 20 s. The supernatants were obtained by centrifugation at 3000 rpm. Rat-specific kits for MDA and GSH measurements were purchased from the manufacturers and the specific methods were followed according to the instructions. The supernatant was analyzed using the commercial kits for MDA (Cat No: YLA0029RA, YL Biont, Shangai, China), GSH (Cat No: YLA0121RA, YL Biont, Shangai, China) and a tissue protein (BR05, Bradford Reagent, EchoTech Biotechnology, Erzurum, Turkey). A microplate spectrophotometer (BioTek, μQuant, Winooski, VT, USA) was used to measure absorbance at 450 nm for MDA and GSH, and at 595 nm for tissue protein.

#### 2.2.5. Histopathological Examination

Lung samples were embedded in paraffin blocks following the fixation in 10% formaldehyde for 48 h by routine tissue processing. Sections of 4 µm thickness were sliced from each block and the preparations prepared for histopathological examination were stained with hematoxylin–eosin (HE) and examined by light microscopy (Olympus BX 51, Tokyo, Japan). Degeneration and necrosis in bronchial–bronchiole epithelium, thickening due to cellular infiltration in interstitial tissues, neutrophil leukocytes in alveolar and bronchiole lumens, and hyperemia severity in vessels were evaluated for histopathological analysis. The sections were graded as none (-), mild (+), moderate (++), and severe (+++) according to their histopathological characteristics.

#### 2.2.6. Immunohistochemical Examination

The deparaffinization and dehydration for the immunoperoxidase assay were performed on the tissue sections of adhesive (poly-L-lysine) slides. Endogenous peroxidase was inactivated by immersion in 3% H_2_O_2_ for 10 min. Tissue samples were then boiled in 1% antigen retrieval solution (citrate buffer (pH + 6.1) 100×) and secured for cooling at room temperature. In order to prevent non-specific background staining of the tissues, the sections were incubated with protein block for 5 min. Then, primary antibodies (Caspase 3 Cat No: ab13847 and JNK Cat No: sc514539, Abcam, Cambridge, UK) in a dilution ratio of 1/100 were applied to the tissues for incubation according to the instructions. 3-3′ Diaminobenzidine (DAB) chromogen was added as the chromogen. The sections were examined under a light microscopy (Zeiss AXIO, Gottingen, Germany) for immunohistochemical evaluation.

#### 2.2.7. Double Immunofluorescence Examination

The same procedures mentioned in the immunohistochemical examination on tissue sections were followed for the deparaffinization, dehydration, inactivation of endogenous peroxidase, boiling in retrieval solution, and prevention of background staining. The primary antibody (8-OHdG, Cat No: sc66036, USA) in a dilution ratio of 1/100 was then applied to the tissues and incubated according to the instructions. Immunofluorescence secondary antibody (FITC, Cat No: ab6785) in a dilution ratio of 1/1000 was used as a secondary marker and kept in the dark for 45 min. The second primary antibody of (H2AX, Cat No: ab20669, USA) in a dilution ratio of 1/100 was then applied to the tissues and incubated according to the instructions. Immunofluorescence secondary antibody (Texas Red, Cat No: ab6719, UK) in a dilution ratio of 1/1000 was used as a secondary marker and kept in the dark for 45 min. DAPI was then dropped onto the sections using mounting medium (Cat No: D1306, UK) in a dilution ratio of 1/200 and kept in the dark for 5 min after covering the sections with a coverslip. The stained sections were examined under a fluorescence microscope (Zeiss AXIO GERMANY).

#### 2.2.8. Western Blot Analysis

Lung tissue samples were stored in a deep freezer at −80 °C prior to Western blot analysis. Lung tissue samples were weighed and processed for analysis. Briefly, the tissue processing was as follows: grounding in liquid nitrogen, treatment with radioimmunoprecipitation (RIPA buffer, Ecotech Bio, Turkey), supplementation with protease and phosphatase inhibitors, and homogenizing for 20 s at 30 Hz with a tissue homogenizer (Qiagen, Hilden, Germany). Lung tissue protein expression was determined using primary antibodies (Table 1). Total lung tissue protein was measured using a protein assay kit (Pierce BCA, Thermo Sci., Rockford, USA). Protein separation (30 µg) was performed by 10% SDS-PAGE and transferred to a PVDF membrane. The blocking of membranes was made with a 5% BSA (bovine serum albumin) at room temperature for 90 min. First, the membranes were incubated with primary antibodies at 4 °C overnight. Then, the PVDF membranes were rinsed with TBST and incubated with the secondary antibody coupled with horseradish peroxidase (Santa Cruz, sc-2004/sc-2005) at room temperature for 90 min. The protein bands were detected by the enhanced chemiluminescence reagent Western ECL substrate (Thermo, 3405). After visualization, the assessments were determined using software (Image Lab™, Bio-Rad, Hercules, CA, USA).

### 2.3. Statistical Analysis

A software package program (SPSS, IBM Inc., Chicago, IL, USA) was used for statistical analysis. The normality test of data was determined by the Kolmogorov–Smirnov test. One-way ANOVA with Tukey’s Post Hoc test was used to compare the data between groups. The Kruskal–Wallis test for interactions and Mann–Whitney U test for differences were used for non-parametric tests. A *p*-value of less than 0.05 was considered statistically significant. To determine the intensity of positive immunohistochemical and immunofluorescent staining, five random areas were evaluated using the ZEISS Zen Imaging Software (V 1.00) program.

## 3. Results

### 3.1. LPS Induction Triggers Severe Sepsis by Elevating Cytokine Levels in the Bloodstream

**Figure 1:**
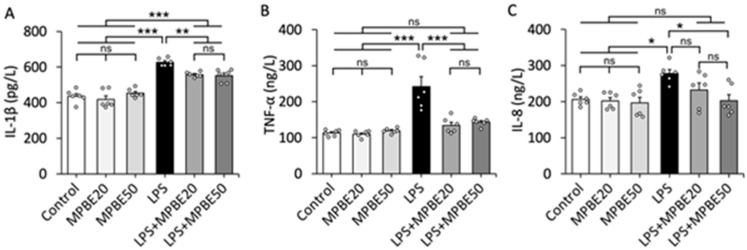
The concentrations of IL-1β (**A**), TNF-α (**B**) and IL-8 (**C**) in the serum. The LPS injection increases the levels of IL-1β (*p* < 0.001), TNF-α (*p* < 0.001) and IL-8 (*p* < 0.05) compared to Control groups indicating sepsis induction. Note that MPBE administration decreases the levels of IL-1β (*p* < 0.01 in MPBE20 and MPBE50 groups), TNF-α (*p* < 0.001 in MPBE20 and MPBE50 groups) and IL-8 (*p* < 0.05 in MPBE50 group) in the MPBE pre-treated septic rats compared to the septic rats without any treatment. Bars are the mean ± SEM. Scatterplots represent the individual data with the exact n number. * *p* < 0.05, ** *p* < 0.01, *** *p* < 0.001, ns: no significant (*p* > 0.05).

The serum concentrations of IL-1β, TNF-α and IL-8 in the groups are presented in Figure 1. There was no difference in serum levels of IL-1β, TNF-α and IL-8 concentrations among the Control, MPBE20 and MPBE50 groups (*p* > 0.05). Intraperitoneal LPS injection increased the serum levels of IL-1β (*p* < 0.001), TNF-α (*p* < 0.001) and IL-8 (*p* < 0.05) concentrations in LPS group compared to the Control, MPBE20 and MPBE50 groups. MPBE (20 mg/kg and 50 mg/kg) administrations decreased the concentrations of TNF-α (*p* < 0.001), IL-1β (*p* < 0.01) in LPS+MPBE groups while 50 mg/kg MPBE administration decreased the level of IL-8 in the LPS+MPBE50 group. Therefore, the increased cytokine levels observed in the septic rats were lowered by MPBE administrations in the bloodstream.

### 3.2. LPS Induction Causes Severe Oxidative Stress in Lung Tissue That Can Be Prevented by MPBE Administration

**Figure 2:**
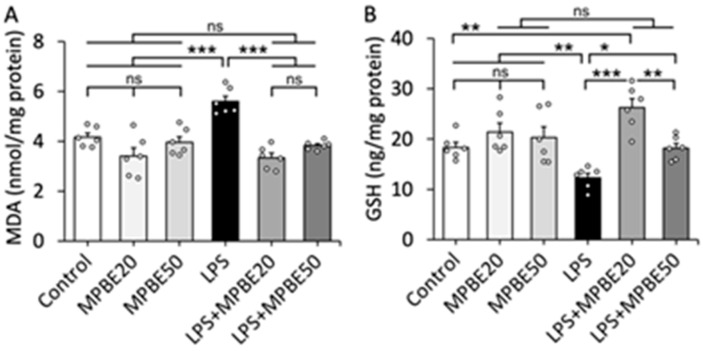
The concentrations of MDA (**A**) and GSH (**B**) in the lung tissue. Oxidative and anti-oxidative status. Bars are mean ± SEM. Scatterplots represent the individual data with the exact n number. * *p* < 0.05, ** *p* < 0.01, *** *p* < 0.001, ns: no significant (*p* > 0.05).

The oxidative stress parameters of the lung tissue are given in Figure 2. The MDA concentration was increased in the LPS group compared to the Control, MPBE20 and MPBE50 groups (*p* < 0.001). MPBE administration decreased the MDA levels in the LPS+MPBE20 and LPS+MPBE50 groups compared to the LPS group (*p* < 0.001). In addition, LPS injection decreased the GSH level in the LPS group compared to the Control, MPBE20 and MPBE50 groups (*p* < 0.01). The GSH level was elevated in LPS+MPBE20 (*p* < 0.001) and LPS+MPBE50 (*p* < 0.05) in the MPBE pre-treated septic rats compared to the septic rats which did not receive any treatment.

### 3.3. Western Blot Analysis Findings

**Figure 3:**
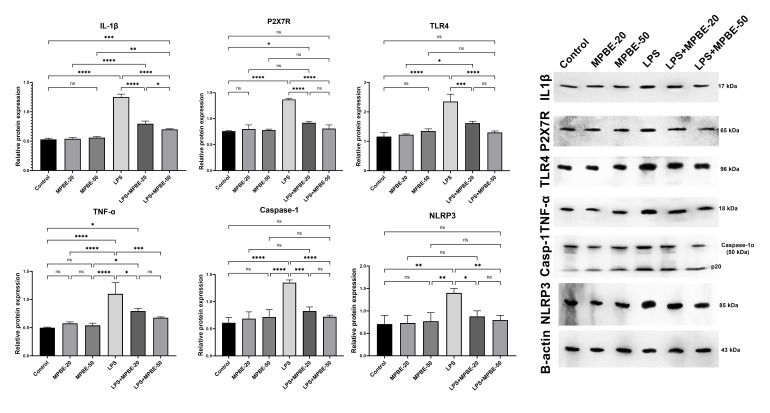
Western blot analysis of IL-1β, P2X7R, TLR4, TNF-α, Caspase-1 and NLRP3 protein expressions in lung tissue of all groups. Data are shown as mean ± SD.Statistical analysis was performed with GraphPad Prism 10.1 using one-way ANOVA and Tukey’s multiple comparison test. * *p* < 0.05, ** *p* < 0.01, *** *p* < 0.001, **** *p* < 0.0001, ns: no significant.

Western blot band images for protein expressions in groups are presented in Figure 3. Evaluation of protein levels obtained from the study tissues revealed statistically significant differences in the expression levels of inflammatory markers (IL-1β, P2X7R, TLR4, TNF-α, Caspase-1, and NLRP3) between the different groups. All proteins were at normal levels in the control group, and MPBE-20 and MPBE-50 treatments did not produce any significant changes compared to the control group (*p* > 0.05). LPS treatment significantly increased the expression of IL-1β, P2X7, TLR4, TNF-α, Caspase-1, and NLRP3 compared to the control group (*p* < 0.0001) and strongly stimulated the inflammatory response. MPBE-20 combined with LPS caused a significant decrease in the expressions of IL-1β (*p* < 0.01), P2X7R (*p* < 0.01), TNF-α (*p* < 0.01) and Caspase-1 (*p* < 0.05) compared to the LPS group, while a partial but not statistically significant decrease was observed in TLR4 and NLRP3 (*p* > 0.05). The LPS+MPBE-50 group significantly suppressed the expressions of IL-1β (*p* < 0.001), P2X7R (*p* < 0.001), TLR4 (*p* < 0.001), TNF-α (*p* < 0.001) and Caspase-1 (*p* < 0.01) compared to LPS, and also provided a significant decrease in NLRP3 levels (*p* < 0.05). Expression analyses and comparisons of all groups and proteins are presented in Figure 3.

### 3.4. Histopathological Findings

**Figure 4:**
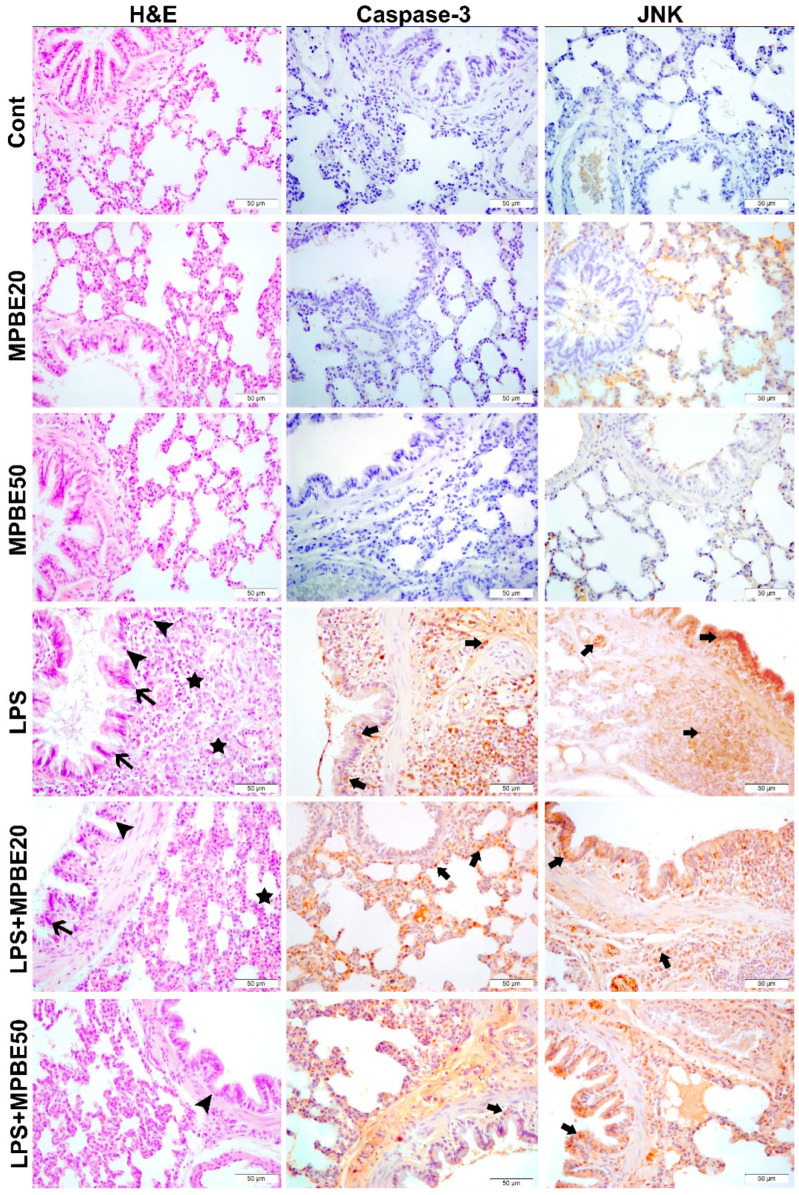
Histopathological appearance of lung tissues in the groups. Lung tissue, degeneration (arrow heads), necrosis (thin arrows) in bronchial and bronchiolar epithelium, thickening and hyperemia due to cellular infiltration (stars) in interstitial tissues, H&E, Bar: 50 µm. Intracytoplasmic caspase-3 and intranuclear JNK expression in bronchial and bronchiolar epithelia, inflammatory cells (thick arrows), IHC-P, Bar: 50 µm.

Histopathological appearances of the lung tissues in the groups are presented in Figure 4. The control, MPBE20 and MPBE50 groups exhibited normal histological tissue appearance without any pathological alterations. In contrast, lung sections from the LPS group showed severe pathological changes, including marked degeneration and necrosis of the bronchiolar epithelium, pronounced interstitial thickening due to intense cellular infiltration, accumulation of neutrophils within alveolar spaces and bronchiolar lumens, and severe vascular hyperemia. In the LPS+MPBE20 group, moderate degeneration and desquamation of the bronchial and bronchiolar epithelium, interstitial thickening, and moderate vascular hyperemia were observed. Notably, the LPS+MPBE50 group exhibited only mild degeneration of the bronchiolar epithelium, with slight interstitial thickening and mild hyperemia, indicating a dose-dependent improvement in tissue morphology.

### 3.5. Immunohistochemical Findings

**Figure 5:**
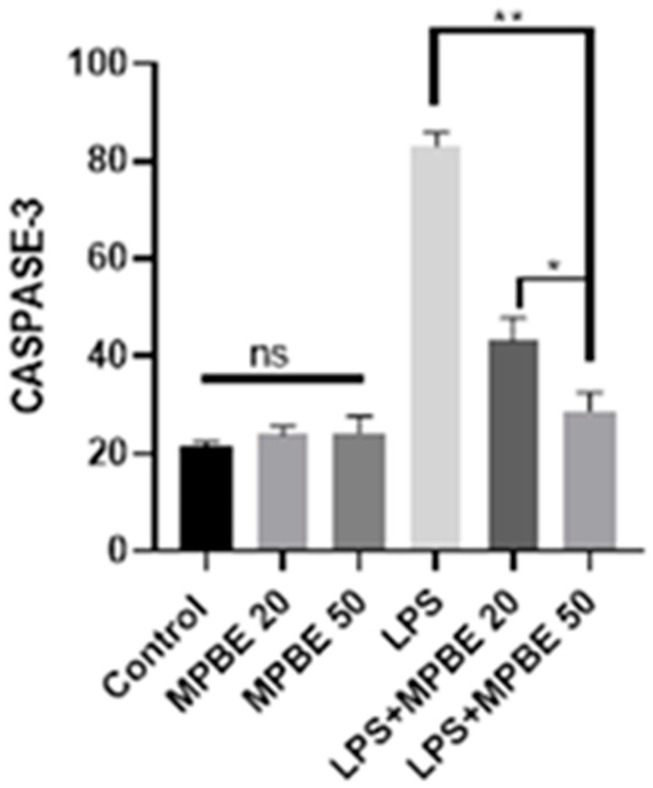
Immunohistochemical staining results observed in lung tissues. Caspase 3 expression levels (* *p* < 0.05, ** *p* < 0.01, ns: no significant *p* > 0.05).

**Figure 6:**
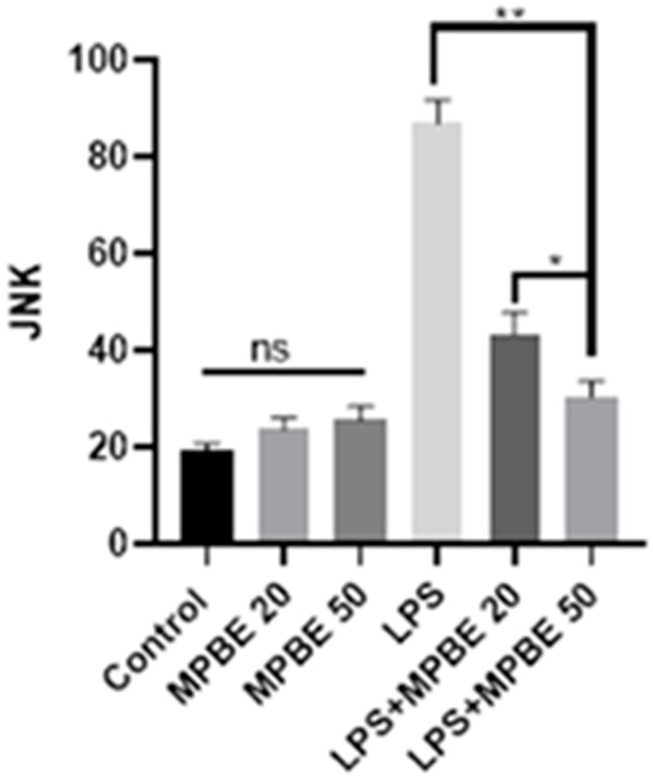
Immunohistochemical staining results observed in lungs tissues. JNK expression levels (* *p* < 0.05, ** *p* < 0.01, ns: no significant *p* > 0.05 ).

Immunohistochemical analysis of lung tissues revealed that both Caspase-3 and JNK expressions were negative in the Control, MPBE20, and MPBE50 groups. In contrast, the LPS group exhibited marked Caspase-3 expression in the bronchial and bronchiolar epithelium, as well as in the cytoplasm of alveolar epithelial cells. Additionally, JNK expression was prominently observed within the nuclei. In the LPS+MPBE20 group, a moderate level of cytoplasmic Caspase-3 and intranuclear JNK expression was detected in the bronchial, bronchiolar, and alveolar epithelia, as well as in macrophages. Notably, in the LPS+MPBE50 group, these expressions were observed with a mild intensity, suggesting a dose-dependent attenuation of apoptotic and stress-related signaling pathways. The findings are presented in Figure 5 and Figure 6.

### 3.6. Immunofluorescence Findings

**Figure 7:**
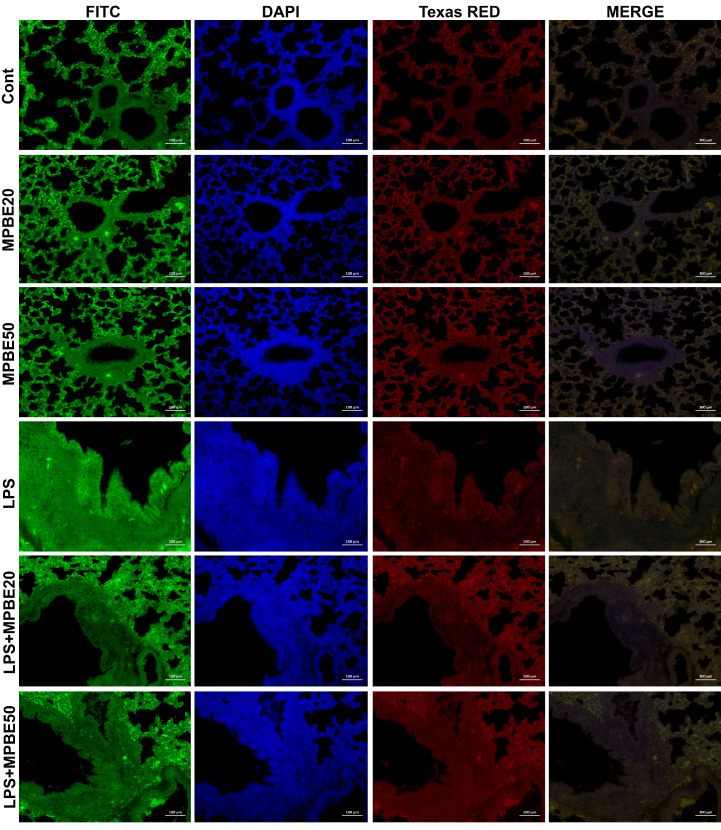
Expressions of 8OHdG (FITC) and H2AX (TEXAS RED) in bronchial–bronchiolar epithelia in lung tissue, IF. Bar: 100 µm.

**Figure 8:**
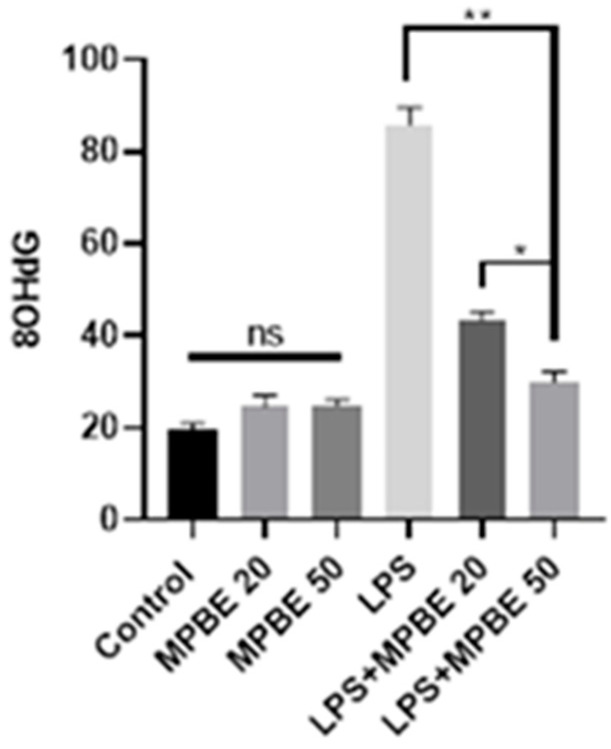
Immunofluorescence staining results of 8OHdG expression levels in lung tissues. * *p* < 0.05, ** *p* < 0.01, ns: no significant *p* > 0.05.

**Figure 9:**
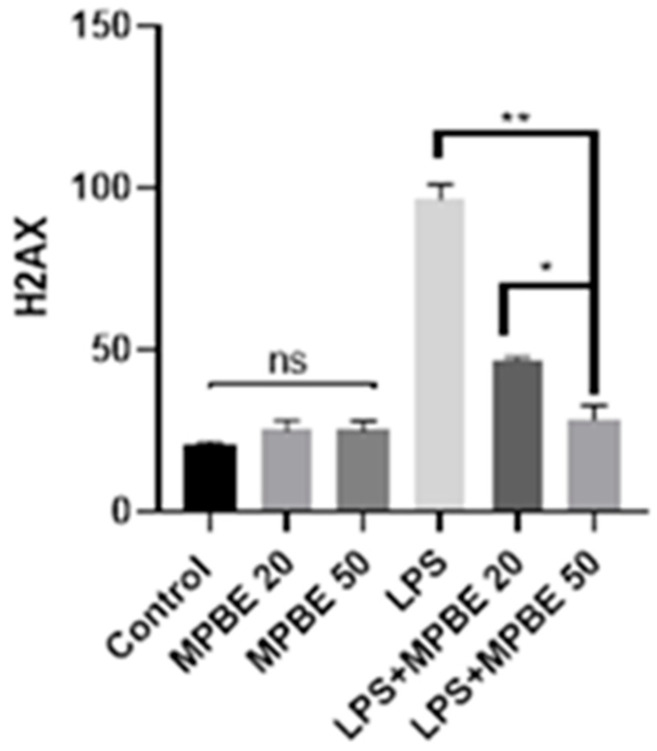
Immunofluorescence staining results of H2AX expression levels in lung tissues. * *p* < 0.05, ** *p* < 0.01, ns: no significant *p* > 0.05.

Immunofluorescence analysis of lung tissues revealed that 8-OHdG and H2AX expressions were absent in the control, MPBE 20 mg/kg, and MPBE 50 mg/kg groups (Figure 7). In contrast, the LPS group exhibited strong immunoreactivity for both markers, with intense expression observed in the bronchial and bronchiolar epithelial cells. In the LPS+MPBE 20 mg/kg group, a moderate level of 8-OHdG and H2AX expression was detected in the bronchial and bronchiolar epithelia. Meanwhile, the LPS+MPBE 50 mg/kg group showed only mild expression of these markers in the same regions, indicating a dose-dependent reduction in oxidative DNA damage. Quantitative data supporting these observations are presented in Figure 8 and Figure 9.

## 4. Discussion

In this study, we investigated the protective effects of per os MPBE (Maritime Pine Bark Extract) administration on lung injury in rats with LPS-induced sepsis. To the best of our knowledge, this study is the first to evaluate the molecular mechanism of MPBE which protects the lung tissue from the damaging effects of LPS by regulating the inflammatory–oxidative–apoptotic pathway associated with purinergic signaling (i.e., P2X7R). Sepsis is a common and deadly syndrome resulting from a dysregulated host response to infection that may lead to ALI and ARDS through overwhelming inflammation, endothelial damage, and pulmonary edema [34]. Lung injury and sepsis have high morbidity and mortality rates, and the current treatment and diagnostic methods for these diseases are not very effective [3]. Given the complex pathophysiology of sepsis involving oxidative stress, systemic inflammation, and multi-organ dysfunction, effective therapeutic strategies such as plant-derived compounds, such as gallic acid [35], *Scutellaria baicalensis,* and *Acacia catechu* [36], have attracted increasing scientific interest. The MPBE is standardized extract rich in polyphenols such as proanthocyanidins, and has broad-spectrum protective effects against oxidative stress, inflammation, and tissue damage [20,22,24,30]. The findings of this study demonstrate that MPBE may alleviate the lung injury caused by LPS-induced sepsis by regulating P2X7Rs, reducing cytokine release and modulating the secretion of inflammatory and apoptotic mediators. These results suggest that MPBE can be used in the prevention and the treatment of lung injury by reducing the inflammatory response of the lung to protect the tissue.

Lipopolysaccharide, an important component of the outer membrane structure of Gram-negative bacteria, triggers the production of various inflammatory cytokines and causes sepsis-induced acute lung injury [37]. LPS can trigger severe inflammatory responses that result in lung injury by interacting with Toll-like receptor 4 (TLR4), which plays a key role in regulating the production of proinflammatory cytokines [38,39]. It has also been reported that LPS increases the expression of P2X7R, which is a strong activator of NLRP3 inflammasome, in lung tissue [16,40]. The formation and activation of the NLRP3 inflammasome can stimulate the production of downstream inflammatory cytokines, including IL-1β and apoptosis-associated Caspase-1, ultimately resulting in pyroptosis [41]. Ding et al. (2025) reported an increase in TLR4, NLRP3, Caspase-1, and P2X7R levels with hypercapnia in mice with LPS-induced sepsis [42]. According to the findings of this study, LPS administration activates the NLRP3-Caspase-1-IL-1β pathway in lung tissue and triggers the inflammatory response by increasing P2X7R expression. In addition, administration of MPBE at doses of 20 and 50 mg/kg significantly alleviates inflammatory changes, suggesting that it may exert an anti-inflammatory effect in lung injury.

Anti-inflammatory drugs could potentially have novel anti-inflammatory and immunosuppressive effects in the treatment of sepsis and septic shock on LPS-induced inflammation and sepsis [43]. The activation of the TLR4 signaling pathway by LPS induction plays a critical role in the regulation of proinflammatory cytokines and mediators. The cytokines exhibit a wide range of proinflammatory functions, such as attracting leukocytes, stimulating phagocytes, promoting the production of downstream cytokines and chemokines, and influencing cell proliferation and apoptosis [44]. Proinflammatory cytokines such as IL-1β, TNF-α and IL-8 begin to be released through an LPS-related pathway that can be downregulated by P2X7R antagonism [16]. In addition, proinflammatory cytokines such as IL-1β are released in response to an infectious disease, while pleiotropic cytokines such as IFN-ω may be used to stimulate the immune response with a promising therapeutic effect [45]. In previous reports, LPS induction exhibited an increase in cytokine levels (IL-1β, TNF-α and IL-8), activating the inflammatory pathway indicating sepsis [16,46,47]. In the present study, MPBE treatment of the septic animals has demonstrated significant reductions in TNF-α, IL-1β and IL-8 levels. MPBE has also been shown to decrease IL-1β and TNF-α in rats with ventilator-induced lung injury [48]. Therefore, MPBE can be used as an anti-inflammatory agent to suppress cytokine production, thus reducing inflammation, and may offer potential therapeutic benefits in inflammatory reactions.

Oxidative stress may also play an important role in the pathophysiology of sepsis. Physiologically, the body maintains a delicate balance between the generation of reactive oxygen species and neutralization through endogenous antioxidant systems [49,50,51]. Studies have indicated that the interaction between oxidative stress and inflammation contributes significantly to the progression of various diseases, playing a key role in the pathogenesis of LPS-induced acute lung injury [52]. In this study, elevated MDA levels and decreased GSH concentrations observed in the LPS-induced sepsis group indicate a pronounced state of oxidative stress. In contrast, rats treated with MPBE exhibited significantly lower MDA levels and higher GSH levels, suggesting a restoration of redox homeostasis. Similarly, the increased expression of 8-OHdG in the LPS group, which reflects oxidative DNA damage, was markedly reduced in the MPBE-treated groups. These findings collectively demonstrate the potent antioxidant capacity of MPBE in mitigating LPS-induced oxidative injury. In accordance with our findings, Taner et al. (2014) reported that administration of MPBE in rats with sepsis significantly reduces the elevated MDA levels while increasing GSH concentration that indicate a clear attenuation of oxidative stress. It has been therefore suggested that MPBE may contribute not only to the prevention of sepsis-induced oxidative DNA damage but also to the enhancement of antioxidant status and activation of DNA repair mechanisms [30].

Caspase-3 and JNK expressions have been found to be increased in the LPS group by the evaluation of immunohistochemistry of the lung tissues in which apoptotic signaling may have been altered by cellular stress. LPS induces apoptosis in the lung through inflammatory pathways and oxidative stress [16,42]. The significant reduction in the apoptotic markers in the MPBE-treated groups, particularly at the 50 mg/kg dose, suggests a dose-dependent anti-apoptotic effect of MPBE, likely mediated by the modulation of signaling and caspase activation. Immunofluorescence analysis reveals a strong 8-OHdG and H2AX expression in the LPS group, reflecting substantial oxidative DNA damage. Treatment with MPBE markedly attenuates these changes, indicating MPBE may enhance DNA stability and repair capacity, possibly by restoring intracellular redox balance and reducing reactive oxygen species. Previous studies also suggest that Pycnogenol (another name for MPBE) enhances antioxidant defenses and mitigates DNA oxidation in models of oxidative injury [30,53]. Thus, Caspase-3, JNK, 8-OHdG, and H2AX strongly support the role of MPBE in protecting lung tissue against LPS-induced apoptotic and genotoxic damage.

**Figure 10:**
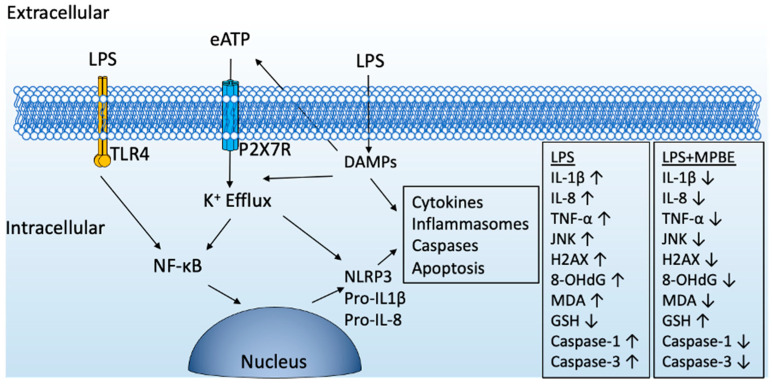
A proposed mechanism of action for MPBE effect in the lung tissue of LPS-induced sepsis.

The administration of LPS significantly upregulates the inflammatory proteins, including P2X7R, TLR4, NLRP3, IL-1β, TNF-α, and Caspase-1 in lung tissue of the septic rats. The upregulation of the inflammatory proteins reflects the activation of TLR4 signaling and the NLRP3 inflammasome pathway, which are essential components in the innate immune response to endotoxin exposure orchestrated by the activation of purinergic signaling. When the involvement of ATP-mediated danger signaling occurs during apoptotic cell damage, the elevation of P2X7R regulates its downstream role in NLRP3 inflammasome activation, ultimately leading to the maturation and secretion of IL-1β and the initiation of pyroptosis [54]. On the other hand, treatment with MPBE at both 20 and 50 mg/kg doses significantly attenuated the LPS-induced overexpression of inflammatory proteins, restoring expression levels compared to the control group. Proanthocyanidins from cranberry extract have been shown to reduce leukotoxin gene expression by suppressing P2X7R and NALP3 gene expression and to neutralize the cytolytic and proinflammatory responses of leukotoxin-treated human macrophages [55]. Therefore, further studies are needed to clarify the molecular mechanisms with regard to MPBE components such as proanthocyanidins and purinergic signaling in sepsis and inflammatory diseases. The attenuation of the inflammatory markers may be indicative of anti-inflammatory activity of MPBE, through modulation of TLR4 and purinergic signaling, as well as inhibition of the inflammasome pathway. Therefore, the downregulation of purinergic signaling and inflammatory mediators clearly confirms the protective effect of MPBE, indicating a potential role in downregulation of inflammatory cascade in sepsis-induced acute lung injury. A proposed mechanism of MPBE effect has been demonstrated in Figure 10.

The histopathological alterations have clearly revealed signs of lung injury during LPS-induced sepsis in this study design. A severe histopathological damage has been observed by the epithelial degeneration and necrosis, interstitial cellular infiltration, neutrophilic accumulation, and marked vascular hyperemia in lung injury in sepsis driven by the intraperitoneal LPS injection. Previous studies have also demonstrated that endotoxin exposure leads to profound inflammatory and vascular alterations in pulmonary tissue [56,57]. The treatment with MPBE has significantly attenuated pathological changes in a dose-dependent manner, as evidenced by reduced epithelial degeneration, decreased interstitial thickening, and milder vascular congestion. The relatively preserved lung architecture in the MPBE 50 mg/kg group suggests that MPBE exherts a protective effect on lung tissue, likely through its anti-inflammatory and antioxidant properties, which contribute to the alleviation of the tissue in lung injury. Therefore, these findings support the potential role of MPBE as a supportive therapeutic agent in the prevention, and possibly in the treatment, of LPS-induced pulmonary damage. However, there may be some limitations for experimental endotoxemia models induced by LPS induction. Animals may exhibit a much higher resistance to LPS-induced endotoxemia, representing some characteristics of the inflammatory response and histopathological alterations. The endotoxemia during live bacterial infection may possibly cause a more severe systemic response than LPS-induced sepsis.

## 5. Conclusions

The findings of this study demonstrate that MPBE exerts significant protective effects against LPS-induced sepsis and lung injury in rats. MPBE effectively alleviates oxidative stress by reducing MDA and 8-OHdG levels while enhancing GSH concentration, and concurrently attenuates inflammatory responses by suppressing the key cytokines such as IL-1β and TNF-α. The critical components of TLR4-NLRP3 inflammasome axis, Caspase-1 and TLR4, thereby regulate the P2X7R signaling pathway and the inflammatory cascade. In response to LPS induction. Histopathological and immunohistochemical analyses further revealed that MPBE protects the lung tissue structure, reducing apoptotic (Caspase-3) and genotoxic (H2AX) responses in a dose-dependent manner. These findings collectively suggest that MPBE may possess anti-inflammatory, antioxidant, anti-apoptotic, and DNA-protective properties, and could potentially be considered as a supportive therapeutic option in the prevention and management of sepsis-induced respiratory complications. Therefore, MPBE administration appears to regulate the inflammatory–oxidative–apoptotic pathway and P2X7R signaling due to its anti-inflammatory, anti-oxidative and anti-apoptotic effects that protect the lung from injury in LPS-induced sepsis in rats, in vivo. In conclusion, MPBE may be a potential therapeutic candidate for the treatment of acute lung injury. Further studies are needed to consolidate and clinically evaluate the data obtained. Specifically, determining dosing protocols to optimize treatment efficacy and examining potential combination strategies should be the focus of future research.

## Figures and Tables

**Table 1:** Primers for Western blot analysis.

Primary Antibody	Manufacturer	Dilution
Caspase-1	Affinity Biotechnology, AF548, Jiangsu, China	1/1000
IL-1β	Affinity Biotechnology, AF5103, Jiangsu, China	1/1000
P2X7R	Santa Cruz, Sc-514962	1/1000
TLR4	Santa Cruz, Sc-293072	1/1000
NLRP3/Cryopyrin	Santa Cruz, Sc-134306	1/1000
TNF-α	Santa Cruz, Sc-52746	1/1000
Beta actin	Santa Cruz, Sc-130065	1/1000

## Data Availability

The data presented in this study are available on request from the corresponding author.

## Abbreviations

The following abbreviations are used in this manuscript:

8-OHdG	8-hydroxy-2′-deoxyguanosine
ALI	Acute lung injury
ARDS	Acute respiratory distress syndrome
ATP	Adenosine 3′-triphosphate
*E. coli*	*Escherichia coli*
eATP	Extracellular adenosine 3′-triphosphate
GSH	Glutathione
IL	Interleukin
JNK	c-Jun N-terminal kinase
LtxA	Leukotoxin
LPS	Lipopolysaccharide
MAPK	Mitogen-activated protein kinase
MDA	Malondialdehyde
MPBE	French maritime pine bark extract
NF-κB	Nuclear factor-kappa B
NLRP3	NOD-like receptor family, pyrin domain containing 3
PAC	Proanthocyanidins
P2X7R	P2X7 receptor
TLR	Toll-like receptor
TNF-α	Tumor Necrosis Factor-alpha
H2AX	Phosphorylated H2A histone family member X
